# Vaginoscopy in Ewes Utilizing a Laparoscopic Surgical Port Device

**DOI:** 10.1155/2017/7404371

**Published:** 2017-09-12

**Authors:** Jeremiah Easley, Desiree Shasa, Eileen Hackett

**Affiliations:** Department of Clinical Sciences, Colorado State University Veterinary Teaching Hospital, 300 West Drake Road, Colorado State University, Fort Collins, CO 80523, USA

## Abstract

Vaginoscopy allows for diagnostic evaluation and treatment of the vaginal vault. A laparoscopic surgical port device and rigid telescope were utilized for serial vaginoscopy in 8 healthy anesthetized ewes. Vaginoscopy examinations were performed in each ewe at days 1, 14, and 28. This technique was well-tolerated and facilitated carbon dioxide vaginal inflation, complete vaginal examination, identification of the cervix, and targeted biopsy collection. No complications were encountered during or following the vaginoscopy procedures. The laparoscopic port device was well-suited to the ewe vulvar size. This technique could be applied to clinical evaluation in ewes for the purposes of examination, biopsy, culture, foreign body removal, and minor surgical procedures.

## 1. Introductions

Vaginoscopy is a useful diagnostic procedure to evaluate the vaginal vault and corresponding structures, such as the cervix, vaginal fornix, and external urethral orifice. It can aid in the diagnosis of genital tract malignancies, foreign bodies, infection, and genital trauma or malformations in both humans and animals [1, 2]. Vaginoscopy is also an effective tool for reproductive management, identifying stages of estrus, predicting optimum stage for breeding, and facilitating transcervical insemination [1, 3]. Vaginal examination has traditionally been performed utilizing a vaginal speculum and light source; however traditional approaches can result in discomfort and are poorly suited for smaller patients or in cases of vaginal stenosis [2]. More recently, vaginoscopy has been performed with flexible and rigid endoscopes, combined with vaginal inflation with gas or fluid, in order to improve examination comfort and efficacy [2, 4].

Single incision laparoscopic surgery has gained popularity and can be facilitated through use of a laparoscopic surgical port device. Port devices have been described in a variety of applications in dogs, including exploratory laparoscopy, ovariectomy, ovariohysterectomy, cryptorchidectomy, gastropexy, splenectomy, and thoracoscopy [5–10]. Port devices can accommodate multiple cannulas and insufflation channels, offering entry points for a variety of instruments through a single port. Because of their flexibility, these devices could be applied to vaginoscopy, allowing simultaneous insufflation, telescopic examination, and insertion of instruments for aspiration, biopsy, or infusion.

The purpose of the present study was to evaluate vaginoscopy in ewes using a laparoscopic surgical port device. We hypothesized that the port device would allow simultaneous insufflation and telescope insertion, resulting in complete visualization of the vaginal walls, identification of the vestibulovaginal junction and cervix, and targeted biopsy procedures.

## 2. Materials and Methods

All research procedures were approved by the Colorado State University Institutional Animal Care and Use Committee (12-3692A) prior to study commencement. Eight mature healthy Rambouillet × Columbia cross bred ewes, approximately six years old and with a mean body weight of 82 ± SD 15 kg, were evaluated as part of a separate study [11]. Food was withheld for 24 hours and sheep were administered phenylbutazone (10 mg/kg per os) and procaine penicillin (30,000 IU/kg subcutaneous) 30–60 min prior to the procedure. Water was not withheld before the procedure. The ear was aseptically prepared and venous and arterial catheters were inserted. Sheep were induced with intravenous ketamine (3.3 mg/kg) and diazepam (0.1 mg/kg) and intubated. Sheep were then moved onto a surgery table in dorsal recumbency and the limbs were secured to the table prior to tilting into a 5-degree Trendelenburg position. Anesthesia was maintained with isoflurane in oxygen and mechanical ventilation, with the % isoflurane administered varying to obtain the appropriate anesthetic depth between 1.5 and 3, and oxygen flow rate of 1.5 L/min. Direct arterial blood pressure was continuously monitored, in addition to electrocardiography, pulse oximetry, and end-tidal capnography. Hypotension was considered in ewes in which the mean arterial blood pressure fell below 65 mmHg and was treated with a single dose of ephedrine between 0.05 and 0.1 mg/kg. All ewes were administered isotonic lactated ringers crystalloid fluids at 3–5 mL/kg body wt per hour IV during anesthesia. Wool surrounding the vulvar region was clipped and the perineum and vulva were aseptically prepared prior to draping the site with sterile towels in preparation for vaginoscopy.

**Figure 1:**
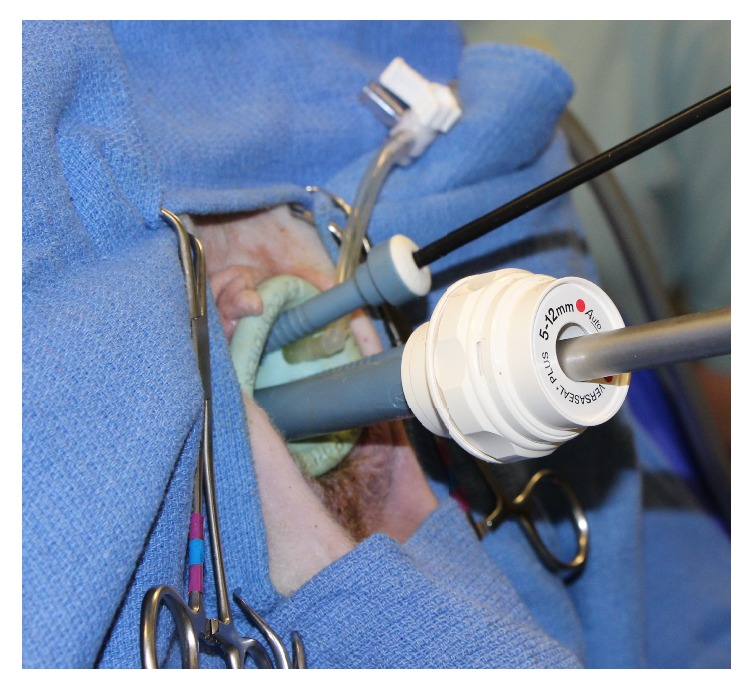
Vaginoscopy is conducted in an anesthetized ewe in dorsal recumbency. The laparoscopic surgical port device is inserted in the vulva, allowing insufflation with carbon dioxide gas and insertion of a 10 mm diameter rigid telescope for complete examination. A 5 mm biopsy instrument has also been inserted through one of the 5 mm portals.

A laparoscopic surgical port device (SILS Port, Medtronic, Minneapolis, MN) was lubricated and inserted into the vagina with curved hemostats until securely in place within the vaginal cavity in all ewes, regardless of variation in body weight. Three cannulas, two 5 mm and one 12 mm, were inserted via the port device. Insufflation tubing was connected from the insufflator (Electronic Endoflator 264305 20, Karl Storz Veterinary Endoscopy-America, Goleta, CA) to the port device for carbon dioxide gas delivery, which was maintained at a pressure of approximately 6 mmHg. A 10 mm diameter, 30°, 33 cm rigid laparoscopic telescope (Hopkins II Telescope 26003BA, Karl Storz Veterinary Endoscopy-America, Goleta, CA) connected to a viewing tower with a camera (Image 1 HD H3 2 camera and 222010 20 processor, Karl Storz Veterinary Endoscopy-America, Goleta, CA) and light source (495NE light cable and Xenon 300 201331 20 light source, Karl Storz Veterinary Endoscopy-America, Goleta, CA) was inserted via the 12 mm cannula within the port device (Figure 1). Upon entering the vagina, visible structures were identified and the examination was video recorded. A 5 mm × 2 mm laparoscopic biopsy punch forcep was inserted through a 5 mm portal within the laparoscopic surgical port device and vaginal mucosal biopsies were obtained from the right and left dorsolateral and right and left ventrolateral vaginal walls. Total procedure duration, including anesthetic induction, positioning, perineal preparation, and vaginoscopy, was recorded.

Following vaginoscopy, sheep were relocated to a recovery area, where heart rate, respiratory rate, position, and swallowing were monitored until anesthetic recovery and extubation. The sheep were reintroduced to feed and maintained in barn confinement for 28 days, with daily monitoring of health.

## 3. Results and Discussion

**Figure 2:**
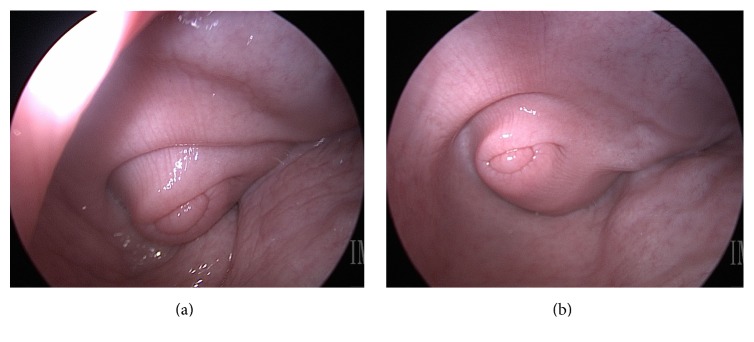
(a) Vaginoscopy image following partial insufflation and expansion of the vaginal vault. (b) Vaginoscopy image following complete insufflation and expansion of the vaginal vault with improved visibility of the cranial vagina and cervix tubercle.

The laparoscopic surgical port device was easily inserted into the ewe vagina and insufflation was maintained via the dedicated insufflation port, despite variation in ewe body weight. A cannula within the port device allowed rigid telescope insertion. The vestibule-vaginal junction, vaginal wall and fornices, and cervix were visible in each sheep evaluated via vaginoscopy facilitated by the port device (Figure 2). Minor manipulation of the laparoscopic telescope visual field and insertion distance was necessary to examine each structure. Insufflation resulted in vaginal expansion and panoramic surface visibility. The right and left anterolateral and right and left posterolateral vaginal walls were easily visualized and biopsies were successfully and accurately performed with minor manipulation of insufflation pressure to reduce tension along the vaginal wall. Three vaginoscopy examinations and biopsy procedures were performed in each ewe for a total of 24 procedures. Hypotension occurred immediately following induction in 7 of 24 anesthetic episodes and corrected within 5 minutes following treatment. Mean procedure duration was 23 minutes ± 4 minutes SD. No secondary vaginal trauma was visible from vaginal examination or port device insertion. Gas vaginal insufflation was released upon removal of the port device. All ewes salivated during general anesthesia and regurgitation was observed in 10% of anesthetic episodes. All sheep recovered well following the procedure, with return to consciousness, extubation, and ability to maintain sternal recumbency within 15 minutes and standing within 30 minutes. No adverse events occurring during the 28-day monitoring period.

Vaginoscopy using the laparoscopic surgical port device was performed in all ewes without procedural or postvaginoscopy complications. Vaginal cavity examination was excellent, compared with previous experience using various specula adapted from other species, due to both vaginal inflation and laparoscopic telescope magnification and illumination. The 30° laparoscopic telescope could be rotated to expand the field of view without losing orientation within the vaginal cavity, similar to previous reports where telescopes with optical angles allowed improved visibility [1]. Vaginal inflation prior to telescope insertion decreases the likelihood of inadvertent trauma to the vaginal wall upon insertion. The rigid telescope could easily be removed, cleaned of mucus, and reinserted without requiring removal of the port device and loss of inflation. The laparoscopic port device was well sized for the ewe vagina and prevented escape of gas insufflation, allowing examination of the vaginal walls, fornices, and cervix, as well as accurate biopsy collection. Inflation of the vaginal cavity did not require addition of balloon dilation or manual compression, which is often necessary with other methods [12]. Image capture for medical recording was also easily performed using this described vaginoscopy method, with image recording preferred to written description for the purposes documentation and evaluating response to treatment.

Vaginoscopy allows inspection of the vaginal cavity, often prompted by abnormal clinical signs of bleeding or persistent vulvovaginitis, and is an excellent diagnostic complement to transabdominal ultrasound examination [1, 12]. Vaginal discharge is a common sign observed in small ruminants with uterine neoplasia and vaginoscopy could be considered to differentiate between or determine the severity of vaginal, cervical, and uterine neoplasms [13, 14]. One benefit of the technique described in the present report is the ease of insertion of examination probes or biopsy forceps through the additional instrument portals in the port device, which could be used to obtain samples for microscopic tissue evaluation and identification of neoplasms. Use of the port device also allows collection of samples for culture, application of topical treatments, and performance of surgery within the vaginal cavity [15]. The laparoscopic surgical port device could be considered for vaginoscopy of animals of similar size, but further validation in other species would be required.

Excellent viewing when utilizing a laparoscopic port device paired with rigid endoscopic equipment for vaginoscopy in ewes could outweigh the associated equipment costs, especially when examinations are performed in hospitals equipped for laparoscopic procedures. In the present report, ewes underwent vaginoscopy under general anesthesia in dorsal recumbency. Ruminants will often regurgitate while under general anesthesia, especially when in Trendelenburg position; therefore fasting prior to the procedure and tracheal intubation with a cuffed tube during anesthesia is necessary to prevent aspiration. Trendelburg positioning in ruminants poses an additional risk of regurgitation under general anesthesia and this informed our use of prophylactic antibiotics. Trendelenburg positioning has been associated with decreased dynamic lung compliance due to increased pressure on the diaphragm and reduction of lung volume, which can contribute to decreased cardiac venous return, cardiac output, and tissue oxygen delivery [16]. Future study is recommended to evaluate the contribution of Trendelenburg position on physiologic parameters in anesthetized sheep. Sedation or other restraint protocols could be considered for ewe vaginoscopy, though further investigation would be needed to evaluate these protocols relative to patient cooperation, ease of examination, and safety [1]. Application of this technique to ewes with vaginal or cervical pathology will be necessary to further investigate its utility in these cases.

## 4. Conclusions

A commercially available laparoscopic surgical port device was successfully utilized for vaginoscopic examination and biopsy collection in ewes. By manipulation of the endoscope and insufflation pressures, it was possible to visualize the cervix and vaginal wall. Thus, application of this technique would be appropriate for complete vaginal examination to detect anatomical alterations, investigate diseases associated with vaginal discharge, and obtain targeted biopsies within the vagina.
